# Forum on immune digital twins: a meeting report

**DOI:** 10.1038/s41540-024-00345-5

**Published:** 2024-02-16

**Authors:** Reinhard Laubenbacher, Fred Adler, Gary An, Filippo Castiglione, Stephen Eubank, Luis L. Fonseca, James Glazier, Tomas Helikar, Marti Jett-Tilton, Denise Kirschner, Paul Macklin, Borna Mehrad, Beth Moore, Virginia Pasour, Ilya Shmulevich, Amber Smith, Isabel Voigt, Thomas E. Yankeelov, Tjalf Ziemssen

**Affiliations:** 1 Department of Medicine, University of Florida, Gainesville, FL USA; 2 Department of Mathematics and School of Biological Sciences, University of Utah, Salt Lake City, UT USA; 3 Department of Surgery, University of Vermont, Burlington, VT USA; 4 Biotechnology Research Center, Technology Innovation Institute, Abu Dhabi, UAE; 5 Biocomplexity Institute and Initiative, University of Virginia, Charlottesville, VA USA; 6 Department of Intelligent Systems Engineering, Indiana University, Bloomington, IN USA; 7 Department of Biochemistry, University of Nebraska, Lincoln, NE USA; 8 U.S. Walter Reed Army Institute of Research, Silver Spring, MD USA; 9 Department of Microbiology and Immunology, University of Michigan, Ann Arbor, MI USA; 10 U.S. Army Research Office, Research Triangle Park, Raleigh, NC USA; 11 Institute for Systems Biology, Seattle, WA USA; 12 Department of Pediatrics, University of Tennessee Health Science Center, Memphis, TN USA; 13 Center of Clinical Neuroscience, Department of Neurology, Medical Faculty and University Hospital Carl Gustav Carus, TUD Dresden University of Technology, Fetscherstraße 74, 01307 Dresden, Germany; 14 Department of Biomedical Engineering, Oden Institute for Computational Engineering and Sciences, Departments of Biomedical Engineering, Diagnostic Medicine, Oncology, The University of Texas, Austin, TX USA; 15 Department of Imaging Physics, The University of Texas MD Anderson Cancer Center, Houston, USA

**Keywords:** Immunology, Medical research

## Abstract

Medical digital twins are computational models of human biology relevant to a given medical condition, which are tailored to an individual patient, thereby predicting the course of disease and individualized treatments, an important goal of personalized medicine. The immune system, which has a central role in many diseases, is highly heterogeneous between individuals, and thus poses a major challenge for this technology. In February 2023, an international group of experts convened for two days to discuss these challenges related to immune digital twins. The group consisted of clinicians, immunologists, biologists, and mathematical modelers, representative of the interdisciplinary nature of medical digital twin development. A video recording of the entire event is available. This paper presents a synopsis of the discussions, brief descriptions of ongoing digital twin projects at different stages of progress. It also proposes a 5-year action plan for further developing this technology. The main recommendations are to identify and pursue a small number of promising use cases, to develop stimulation-specific assays of immune function in a clinical setting, and to develop a database of existing computational immune models, as well as advanced modeling technology and infrastructure.

The concept of a *medical digital twin* (MDT) represents a pivotal technology envisioned to make personalized medicine a reality. This entails using predictive computational models to harness diverse patient data over time, allowing for identification of optimal interventions and corresponding predictions of their effectiveness for an individual patient; see ref. ^1–4^. Scaling up this concept into a widely used medical technology necessitates substantial coordinated advancements across several fields, including human biology, medicine, biochemistry, bioinformatics, and mathematical and computational modeling. A sign of increasing interest in this technology was evident in the workshop “Opportunities and Challenges for Digital Twins in Medicine,” organized by the National Academies of Science, Engineering, and Medicine in January 2023^5,6^. One possible long-term vision is a virtual replica of an entire patient that evolves with the patient over the course of their lives, as articulated by the Virtual Physiological Human Institute^7^ and the European Virtual Human Twin Project^8^. The foundations for MDT technology, however, are yet to be developed. The Forum described here, and other efforts^9,10^ have focused on digital twins for medical conditions related to the immune system. This provides a narrower focus, but at the same time addresses a wide range of diseases that involve the immune system in an essential way, such as infectious diseases, autoimmune diseases, and cancer, among many others. The immune system in many ways serves as a benchmark for the kind of complexity that we need to be able to represent with computational models. High-fidelity models might be needed to account for differences in an individual’s immune response to, say, a vaccine. One desired outcome is a broad-based community effort to advance modeling of the immune system in different disease contexts.

We adopted a broad definition of a MDT that happens to align with that in a recently released report by the National Academies of Science, Engineering and Medicine on “Foundational Research Gaps and Future Opportunities for Digital Twins”^11^: “*The key elements that comprise a digital twin include (1) modeling and simulation to create a virtual representation of a physical counterpart, and (2) a bidirectional interaction between the virtual and the physical. This bidirectional interaction forms a feedback loop that comprises dynamic data-driven model updating (e.g., sensor fusion, inversion, data assimilation) and optimal decision-making (*e.g., *control, sensor steering)*. A key point is that there is an ongoing exchange between the patient and the digital twin, with data from the patient used to dynamically recalibrate the digital twin, and predictions from the digital twin informing patient treatments. While there are many computational models of human biology in the literature that could be further developed into MDTs, specifying, and incorporating the ongoing bidirectional data links between individual patients and personalized computational models has not been fully explored in most cases, so that extensive further development is required. The Forum, the subject of this report, was primarily focused on such early-stage MDTs and what is needed to progress to clinical applications.

An international group of experts convened in Lake Nona, FL, February 23–24, 2023, for the “Forum on Precision Immunology: Immune Digital Twins”^12^, supported by a grant from the Biomathematics Program at the U.S. Army Research Office. The aim was to discuss these questions and assess examples of ongoing modeling projects that are part of MDT development related to immunity. This report encapsulates a synthesis of these discussions and an outline of challenges to be addressed over the next five years. The development of MDTs takes place at the interface of medicine, experimental biology, and mathematical modeling (see Fig. 1). The Forum participants are all authors of this article, and represent a cross-section of these fields, including immunologists, clinicians, experimental biologists, and mathematical modelers. The Forum served as a venue to discuss the different perspectives each of these communities has on the prospect of using personalized computational models in the clinic. To facilitate an exchange of ideas across these fields, the program consisted of a collection of 45-minute blocks, with a 15-minute presentation by a participant, followed by 30 min of discussion. The only audio-visual aid available to presenters was a whiteboard, favoring discussion over formal presentations. High-quality audio-visual recordings of the individual sessions are available through links at^12^. A preliminary version of this manuscript is part of the preprint “Forum on Immune Digital Twins: A Meeting Report,” by the same group of authors^13^. The reader is encouraged to view the presentations, as they contain many valuable ideas, viewpoints, and information not contained in this synopsis. In fact, we believe that the true value of the workshop lies in the extended discussions between participants of different backgrounds that explored a wide range of complexities not easily done in more conventional formats. This was the motivation for the recordings capturing all participants and the whiteboard, with high-quality audio. This report does not do justice to the depth of the discussions. On the other hand, the recordings also reveal the limitations of the Forum. Given the small number of participants, the content is necessarily limited in its breadth and comprehensiveness, even though it was already narrowed to issues related to immunology.

**Fig. 1:**
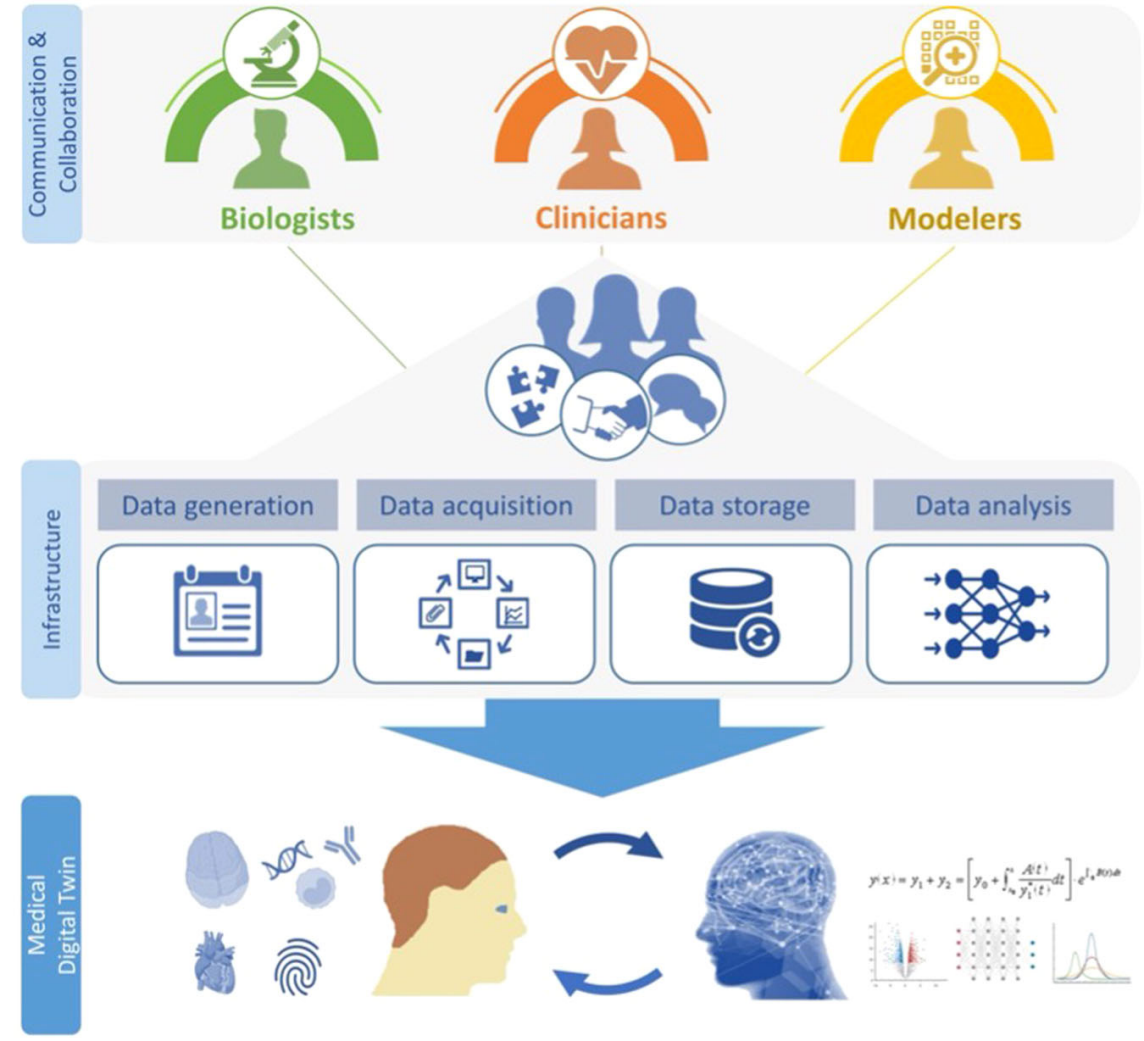
MDT Development. The development of MDTs requires the collaboration and close communication of three communities: biologists, clinicians, and computational modelers. They are connected through an infrastructure that allows the flow of data and information connecting the digital twin with the patient. The end result is a digital replica that closely tracks the patient over time.

We now outline the general themes of the Forum discussions for each of the three pillars of MDTs: human immune system biology, the clinic, and mathematical and computational modeling. And we extract a collection of action items for a 5-year plan to further MDT development.

## Human immune system biology

The human immune system is highly specialized and has evolved to have exquisite specificity for defending its host from injury and infection. During health, the immune response is tightly orchestrated to respond to threats without inducing significant tissue damage, but dysregulation can occur, contributing to cancer or autoimmunity^14^. The complexity of the immune system is such that attempting to explain it is the subject of a highly-circulated joke^15^. However, we believe that our goal creating MDTs involving the immune system provides some guidance as to how to practically address this complexity: specifically, since the transition from one data collection point to another is intrinsic to the concept of an MDT (*e.g*., dynamics) this allows for an explicit focus on characterizing function(s) of the system versus merely descriptive system state characterizations. Consider the following sources of complexity and associated challenges at multiple scales for obtaining predictive functional immune assessment from more classical immune state descriptions:

### Genetic and molecular interactions

A classical way of characterizing the adaptive immune response in any individual is to describe the ability of the host cell to display peptides derived from foreign threats (*e.g*., microbes) in the context of human leukocyte antigens (HLA). Every person inherits 6 major HLA alleles from each parent (HLA- A, B, C, DP, DQ and DR for 12 total) and there are over 37,000 HLA and related alleles characterized to date^16^. However, while there are good computational tools to predict what peptide will fit into the groove of the appropriate HLA molecule of each person^17^ we do not yet have good methods to predict which of the many possibilities is likely to be the predominant driver of an individual’s immune response, *e.g*., the immunodominance of the response to exposure across a set of antigens. Immunodominance is related to the cadre of T and B cell receptors that are present in each individual, where it is estimated that 10^13^–10^18^ different T and B cell immune receptors are generated through genetic rearrangements, reassortments and editing, with many of the cells carrying autoreactive receptors (or those that don’t recognize self at all) deleted during development, leaving approximately 10^11^–10^12^ different specificities in circulation^18^. However, while there has been considerable progress in the development of machine learning and artificial intelligence algorithms that can predict peptide binding to HLA, T cell binding to peptide-HLA and T helper cell differentiation programs^18–22^, the means of predicting the manifestation of immunodominance in response to a specific antigen remain insufficient. This is due, in great part, to the multiscale nature of the immune system, which is a recurrent theme regarding the challenges we face.

### Cellular interactions

The molecular events previously described are distributed across cellular populations, and this encapsulation of the data regarding binding affinities and their subsequent consequences represents the next scale of immune complexity. The ability of a particular T cell to encounter a particular antigen-presenting cell with the correct HLA and the correct peptide for activation is stochastic in nature, involving the probability of the two cells encountering one another and the strength of the interactions and co-receptor signaling to activate the cells. Furthermore, once activated, how individual cells respond to differential mediator milieus adds a further level of complexity regarding how T cells differentiate into particular subsets with unique attributes^23^.

### Tissue/Systemic factors

At the next scale, all the above factors are subject to differences in the relative concentrations and types of immune cells and respective target antigens in the specific tissue of engagement, the effect of a particular tissue milieu on motility factors, and the pre-existing systemic immune milieu of an individual patient (*i.e*., affected by drugs, prior infections, co-morbidities, age, etc.). Systemic metrics take on extra importance because currently blood/plasma represents the most readily accessible sample source by which patient state is measured. This situation presents two distinct challenges. The first is obtaining sufficiently broad and rapid assays to reflect the multi-parameter complexity of the immune mediator milieu; current methods such as Ella^24^ provide a starting point for further development. The second is the realization that very often there is a discrepancy in the immune dynamics in a specific tissue and that reflected by circulating mediators/cells. This latter issue will require developing novel approaches to interrogate, in a non-destructive fashion, the cellular and molecular processes within specific tissue/organ compartments.

In addition to the inherent structural complexity of the immune response there needs to be a recognition of the importance of stochasticity in biological systems operating at multiple scales. While patient heterogeneity can be invoked in clinical settings, even in the research laboratory where experiments can be conducted in genetically identical individuals (*e.g*., mice), housed in identical conditions and infected with identical pathogens and doses, we see variations in the response. For example, for any infection, researchers can generate an LD_50_ which is defined experimentally as the dose at which 50% of the animals die; what then are the factors that lead to this discrepancy in outcome? This heterogeneity is also present in in vitro monocultures of clonally-generated cells, where a distribution of responsiveness to a uniform experimental perturbation is near ubiquitous. Given that multiscale stochasticity is evident throughout the immune response in disease, mechanisms and methods for incorporating stochasticity need to be incorporated into the design of an MDT.

Thus, the sheer complexity in terms of genetics, random interactions, cytokine profiles, receptor diversity and outcomes are daunting when considering how to model individualized immune responses within an MDT. However, we believe that all is not lost: as noted above we believe that the recognition that what is important in achieving the MDT paradigm provides an opportunity to shift how we characterize the immune system in terms of functional responsiveness. For example, the current paradigm of identifying immune subset phenotypes utilizes RNA sequencing technology and multi-parameter flow cytometry (with flow cytometry being a potentially real-time source of data) to sort cells by cell surface receptor status, but we know the molecular phenotype of the cells does not necessarily connote function. For an MDT it is less important to know how a specific cell type is defined than it is to know what *the cell type does within a specified disease context*. For example, current functional cell response assays (*e.g*., ELISpot) have been used to identify potential paralysis or exhaustion in immune cell subpopulations^25^. We believe that these are the type of technologies that can be built upon to develop rapid functional assessment of a desired cellular behavioral output (*e.g*., production of interferon gamma in response to a viral antigen stimulation). In modular immune digital twins, this information can be used to monitor/feed-back on computational immune subcomponents in a bidirectional fashion while tracking and controlling a disease trajectory. We pose that developing a repertoire of critical functional assays for a specific disease process will be an effective way to aggregate the many factors and features of immune activation into a tractable set of variables for modeling immune response with an MDT.

#### Five-year action plan for human immune system biology

Given the multiscale nature of the immune response in disease, our action plan for the next 5 years proposes targets at multiple levels, with the overarching goal of collapsing an immune potential (which would be defined across a population and is how the immune system is currently characterized) into an individualized trajectory of a specific patient at a specific time to which the MDT is “twinned”.

1. In terms of adopting a more functional view of the immune system, a major goal should be the development of enhanced in vitro and ex vivo assays to accurately predict immune function of selected cellular populations in response to both endogenous mediators and clinically relevant pathogens. This development should ideally be explicitly translational, seeking to take methods available at the experimental laboratory level (such as cell sorting and stimulation-response experiments) into clinically deployable assays (such as ELISpot).
2. Of note, the development of such assays would provide the opportunity for personalized immune state characterization; the profiling of an individual’s immune cell subpopulation responsiveness to a set of defined stimuli would serve as a “baseline” for ongoing monitoring and early warning (in addition to providing disease trajectory identification in the acute setting).
3. From a system-level monitoring standpoint, these developments would be complemented by refinement and expansion of multiplexed assays with clinically relevant turn-around times. There is also the potential to develop personalized multi-cellular organoid/organ-on-a-chip systems that can be used to determine responsiveness to potential interventions.
4. The creation of multi-scale “maps” between cellular-molecular configurations and clinically available physiological data. The creation of data sets of such maps will be critical in being able to leverage machine learning/artificial intelligence methods to phenomenologically link patient data across scales. There is a potentially critical role for the ability to generate biologically-realistic synthetic data in order to augment what will be invariably sparse clinical data^26^. We recognize that the development of these capabilities is extremely ambitious. However, this is where we envision an important role for MDTs: to identify the requirements needed to actually deploy an Immune MDT (and thereby gain the benefits of digital twin technology), which can guide and focus research towards providing the necessary types of data-generating sensor technologies. This is particularly crucial given the intrinsically dynamic nature of MDTs, with an emphasis on needing to represent functional phenotypes versus generating “list-of-features” descriptions of biological entities.

## The clinic

The “twin” component of an MDT explicitly ties the digital object to an individual patient, and therefore inherently incorporates a translational purpose of the MDT. As such, the potential clinical role of an MDT will drive its development. Clinical practice can be divided into a series of distinct, but related tasks: 1) diagnosis of a potential disease state (this includes monitoring a state of health to identify divergences); 2) prognosis, which attempts to predict or forecast a particular disease trajectory; 3) personalization/optimization of existing therapies; and 4) the discovery/testing of novel treatments. Items 1–3 form the basis of current clinical practice, with a mixture of basic pathophysiology, evidence-based (ideally) practice guidelines and an individual physician’s expertise and intuition. Conversely, Item 4, the discovery/testing of novel treatments, is traditionally the purview of research. These tasks can also be grouped into types: 1) a classification task (“What illness is the medical team dealing with?”); 2) a forecasting task (“What is going to happen to my patient in the future?”); and 3) a control task (“What is the best course of action to make my patient better?”). Classifying a particular use-case for potential MDTs can aid in determining what sort of data is necessary and available (or not) for a particular purpose, what the time scale might be for the updating between the MDT and the real-world twin, and what type of computational method(s) would be needed to propagate the MDT forward in time (this aspect will be covered in more detail in the “Mathematical and Computational Modeling” section below). Another application one could envision is for an MDT to serve as a benchmarking tool to evaluate current therapy.

As an example, we present a potential use case of MDTs in the treatment of sepsis, one of the largest sources of morbidity, mortality, and health care costs world-wide (WHO). The unfortunate fact of sepsis is that, to date, there is no generally accepted means of interrupting the underlying inflammatory/immune biology that drives sepsis and its subsequent organ failure. Major contributing reasons for this are the overall heterogeneity of the septic population (reflected in a gap between the means of “diagnosing” sepsis and the degree of knowledge regarding the cellular-molecular mechanisms that drive the disease) and the complexity, both in terms of the underlying biological mechanisms and their dynamics in given different insults, of the disease course. In short, effective treatment/control requires identifying the right patient at the right time for the right set of therapies, and the current means of doing these tasks for a septic patient are woefully inadequate. It is here that MDTs can play an invaluable role in personalizing the characterization of a septic patient so that “right patient, right time, right drug(s)” can be achieved.

We briefly mention one other use case presented at the forum, and refer the reader to the Forum recordings^12^ for this and other examples. The MDT project described in^27^ predicts the progression of breast tumors using a partial differential equations model of breast tissue. The model is personalized to a particular patient by using patient-specific images from both MRI and quantitative positron emission tomography. Model parameters that capture cell migration and tumor cell proliferation properties of the specific tumor are derived from image analysis. The resulting digital twin can be used to predict the efficacy of certain drugs, as well as immunotherapy^28^.

A key feature of an MDT is its connection to the physical patient through the periodic recalibration of the model with data collected from the patient. For applications involving the immune system, it is unlikely at the present time that data can be collected at near streaming rates, like for vital signs or fitness trackers. Currently, the most common means of repeated data collection, in addition to patient records, is through analysis of fluids collected from the patient, such as blood draws, bronchoalveolar lavage, urine, etc. Another data source is imaging data such as x-rays or CT scans. Data could also include sequencing data collected on a limited basis. There are many well-known issues related to the quantity and quality of such data, such as standardization, and irregularity of data collection. These issues are more easily addressed in the context of clinical trials, for instance, than for regular hospital operations. And, of course, there are significant privacy issues attached with data collection and transfer that are only beginning to be considered, as part of efforts such as^8^.

### Five-year action plan for the clinic

A Five-Year plan for the development and deployment of MDTs needs to integrate capabilities that can improve patient health, with aspirational capabilities that will allow MDTs to reach their full potential within a decade. With this in mind, we propose the following actions:

1. Explicit definition of specific use-cases/tasks for a given disease.
2. Identification of specific data types required for each use case, whether that data currently exists in some form or will be available in the future to meet the capabilities of an aspirational MDT. Of note, obtaining time series/ongoing collected data is essential to this step, as the concept of time-evolution of the MDT is inherent to its definition.
3. These first three steps should be integrated into a detailed “roadmap” for the development and deployment of the MDT.
4. Use of this roadmap to engage collaborators and stakeholders (*i.e*., immunologists, clinicians and clinical researchers, assay developers, mathematical modelers, and biologists) to facilitate the collection of existing data and develop the capability to acquire new types of data as needed.
5. Deployment of an initial MDT with diagnostic and prognostic utility for clinical decision-support. Ideally, there should be enough preliminary data such that reasonable planning could be implemented after a short period for clinical trials to demonstrate their utility.

## Mathematical and computational modeling

**Fig. 2:**
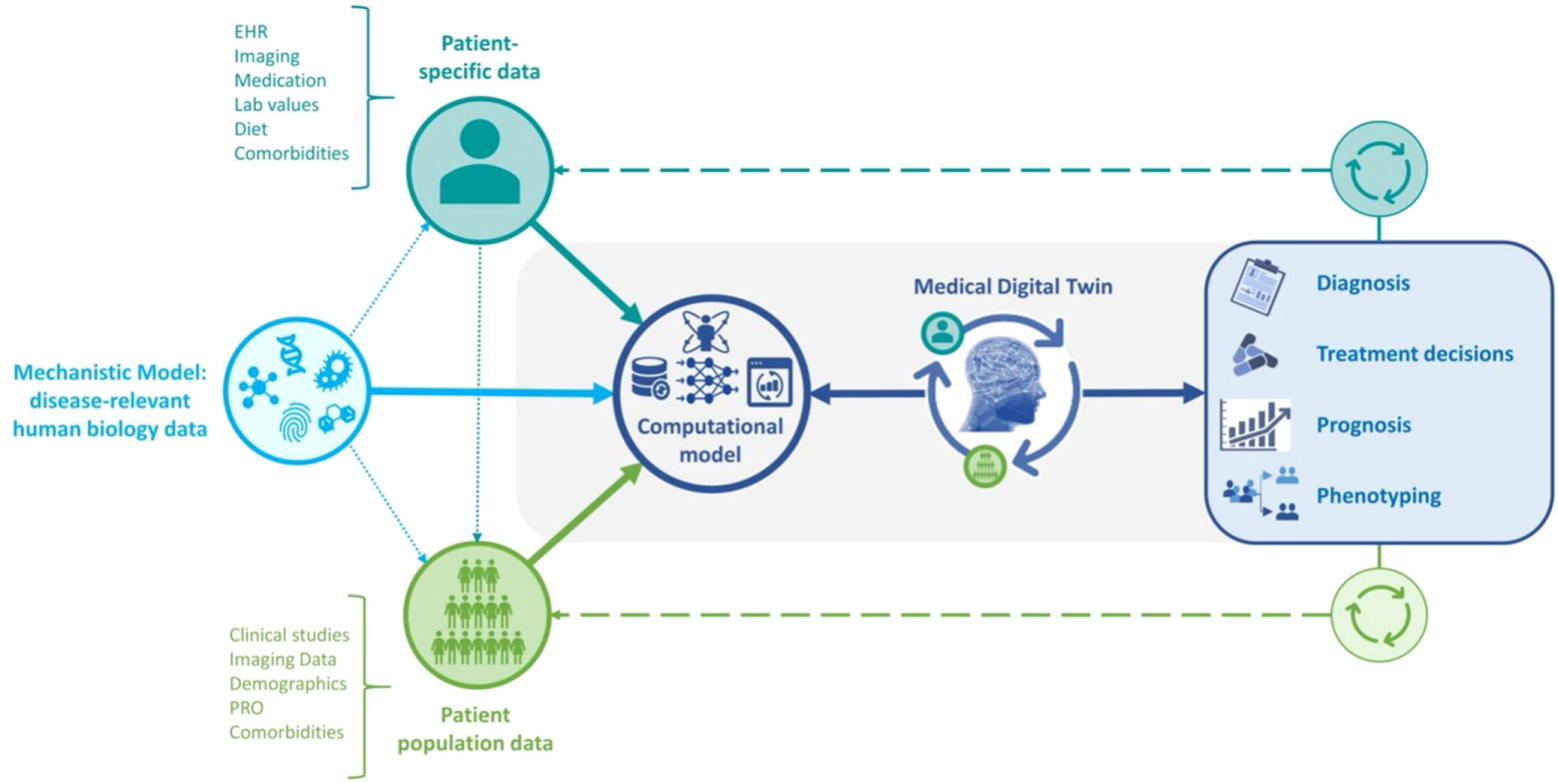
Models Underlying MDTs. The computational model at the heart of an MDT serves several purposes. It integrates human biology, clinical data, data characterizing reference populations, and patient-specific data. It is personalized to the patient and is periodically re-calibrated. Control algorithms attached to the model can be used to optimize available patient treatments.

The engine of any MDT is a computational model. Depending on the application and available data, it may include mechanistic information about the relevant human biology, and it may take as input information specific to either an individual patient or a patient population. In all cases, the output is information that can be used in the treatment of an individual patient. Figure. 2 depicts the role of the computational model in the workflow of MDT applications.

For instance, a deep learning model might be trained on clinical data from a large patient cohort of gastric cancer patients, and is then used to determine a patient’s response to an immunotherapy treatment^29^. Such models may or may not include any mechanistic information about the relevant tumor biology, such as mutated signaling pathways and their downstream effects, and predictions for the specific patient are based on correlation between the patient’s data and those of the reference population used in the model. At the other end of the spectrum, a computational model may capture all known features of human biology relevant to a given application and may make treatment recommendations based on a model analysis, informing clinical trials, without directly using any data from a specific patient^29^. The focus of Forum participants was primarily on MDTs based on a mechanistic computational model. This preference stems from the ability of mechanistic MDTs to link outcomes to mechanisms, thereby informing treatment. Additionally, these models allow for the performance of uncertainty quantification in relation to their predictions.

Many mechanistic models of human biology are now available, particularly those incorporating aspects of the immune system. For numerous applications, the underlying model of an MDT will need to encompass various mechanisms, spanning several spatial and temporal scales. For example, while most drug mechanisms are intracellular, their effects manifest at the tissue or organ scale, necessitating cross-scale integration. The immune response to an infection is multifaceted, coordinating diverse mechanisms and cell types. Consequently, computational models for MDTs will likely be high-dimensional, multi-scale, multi-physics, hybrid, and stochastic, containing numerous parameters. Integrating heterogeneous data types, from molecular to physiological, will be essential for their parameterization and application. Most crucially, these models should be adaptable to individual patient data. Very few such models have been constructed for clinical use or new biology discovery, leading us into uncharted territory in their construction, analysis, validation, and application.

An important issue in quantitative medicine in general and MDTs in particular is data integration. We first note that MDTs are an ideal vehicle for the integration of heterogeneous data types at different scales, from molecular to organism-level data, since they provide a rigorous framework that links heterogeneous data types characterizing heterogeneous biological processes in the correct biological fashion. Practically, however, this can raise many challenges, unique to different applications. There are no general approaches to this problem, to our knowledge. In addition, data collection might be technologically challenging. Sometimes, surrogate data types can be used. For instance, gene expression data are often used as surrogates for protein data, even though it is well-understood that this is often problematic for several reasons, *e.g*., lack of correlation in expression. Also, while it is sometimes possible to obtain data at different scales from the same experiment, this is not always the case.

Finally, we comment on the use of machine learning and artificial intelligence (ML/AI) models for the purpose of constructing MDTs. For the purpose of illustration, we focus on cancer applications, since this is a field that is comparably rich in data. It is important to note that there are certain problems of central importance to oncology where the methods of AI/Big Data are fundamentally limited in their ability to power digital twins. For example, predicting treatment response and then, subsequently, identifying an optimal interventional plan for an individual patient. Examples of the need to solve this problem abound in both medical and radiation oncology where it is well-recognized that a “one-size-fits-all” approach is not appropriate, but a practical method for identifying optimal patient-specific interventions is not well-established. Given the tremendous heterogeneity between patients, it is difficult to imagine how a digital twin built on population data can provide anything more than general insights into predicting an individual patient’s response, let alone how to optimize their treatment plan^30^. This is because a patient is not just diagnosed with cancer, or (for example) breast cancer, or (for example) triple negative breast cancer; rather, they are diagnosed with one of the (currently) known subtypes of triple negative breast cancer. Thus, to build a digital twin for Ms. Jane Doe that is powered by population-based data, one would need to find a training data set with hundreds (thousands?) of patients that share her subtype, her biological characteristics, and contains all the therapeutic regimens she might receive. That data set does not exist and is unlikely to ever exist because cancer is getting more precisely diagnosed (thereby increasing patient heterogeneity) and the number of available drugs is increasing (thereby increasing treatment options). (This, in fact, recently played out for early triple negative breast cancer when pembrolizumab was approved as part of the standard-of-care therapy^31^, thereby necessitating building new databases for all population-based approaches for this disease). This is but one example of a problem for which biology-based mathematical models offer a distinct advantage over the AI/Big Data only approach. By explicitly including known biology and physics into the mathematical model^27^, one can calibrate such models using only patient-specific data to personalize the digital twin, thereby allowing one to not only systematically simulate patient-specific interventions, but also select the one with the highest probability of yielding a positive outcome^32^.

### Five-year action plan for mathematical and computational modeling

1. The biomedical modeling community has spent decades building complex models of different medical and disease processes in humans from cancer to infections. These are all potentially usable as drivers of MDTs or components thereof. As a first step, we need to develop and curate a repository of model templates (*i.e*., accepted model structures) and specific model models (*e.g*., peer-reviewed models of specific signaling networks) that can be used in the construction of MDTs, ranging from intracellular to physiological scales. Existing repositories include, *e.g*., Biomodels^33^, Cell Collective^34^ and GinSim^35^. These can be built upon for a more comprehensive curated collection.
2. Existing techniques for the validation, calibration, and analysis of computational models, most importantly sensitivity and identifiability of model parameters, are not always directly applicable to models underlying an MDT. Research is needed to develop appropriate model analysis techniques for MDTs.
3. For many applications, MDTs will be used to forecast the future health trajectory of a patient, as well as the effect of available interventions to change it. Existing control and optimization methods (e.g., ref. 28) mostly apply only to ordinary differential equations models. Research is needed to develop novel forecasting and control approaches suitable for complex MDTs.
4. There are many existing models of disease processes and immune system function that can be used to build MDTs, as mentioned above. Research is needed to develop a platform for the modular construction of complex MDT models from component models. Such a platform is essential for achieving the long-term vision of a virtual patient. A possible approach is proposed in^12,36^.

The data from an individual patient captures different aspects of their characteristics and health status. We have genomic data, gene expression measurements, protein, and metabolite concentrations in different tissues under different conditions, imaging data of everything from immune cells in lymph nodes to functional MRI data in the brain, electronic health records, to lifestyle and behavioral data. They all provide information about some aspect of a person, and the challenge is to integrate them in a meaningful way to provide a holistic representation. A computational model of the patient that is dynamically updated with all this information is a natural way to accomplish the data integration required. The confluence of several simultaneous developments has created an environment in which this promise of personalized medicine is taking on shape: vastly increased availability of data, from the molecular to the population scale, leading to a deeper understanding of human biology and its role in health and disease, and, finally, an expansion of our computational and modeling tools.

As mentioned earlier, most of the projects discussed at the Forum do not meet the criteria for being considered a digital twin or being readily turned into one. All the models discussed can, in principle, serve as the basis for a computational model that is personalized to a patient as all of them capture some aspect of disease-relevant human biology. The most common reason this has not been done for the models discussed is that they are still in the phase of model validation using, in most cases, animal or in vitro data, as appropriate human data are often not available. The second reason is that patient data routinely collected in a healthcare setting are often not suitable to be used directly for the calibration of the models discussed, since models often contain events at the intracellular scale or spatial heterogeneities that are difficult to capture at the tissue scale. Thus, the first step needs to be to develop surrogate measurements for unavailable data and to develop surrogate models that can be used as the underlying MDT model.

If the experience of the computational biology community over the last 30 years is any indication, then possibly the most daunting challenge to widespread adoption of the MDT paradigm is the formation and functioning of the interdisciplinary teams required for this purpose. In addition to biologists and mathematical modelers, we need to also integrate clinicians. All three communities need to find ways to come together for both research and deployment of this technology. This point is made strongly in the National Academies report on the subject^11^. The Forum participants included representatives of the three communities, and the difficulty of aligning objectives and bridging language barriers was discussed. There are no ready-made solutions to this problem, but appropriate funding mechanisms requiring this integration can provide incentives.

A well-designed funding program for MDT research by the public sector is crucial if substantial progress is to be made over the next decade. New funding paradigms should be considered for this purpose. There can also be an important role for the business community and philanthropic organizations in providing funding for this effort and collaborating on the myriad research problems that will need to be tackled and solved. The Forum we are reporting on here is intended to support a dialog around this topic. Collectively, a community is emerging around this effort that can, with the right resources, help make rapid progress on bringing MDTs to patients at a large scale.

Finally, we comment briefly on a number of important issues related to MDTs that were not discussed at the Forum, in part because time was very limited, and in part because the topics required expertise not represented among a significant number of the participants. This includes regulatory hurdles to the deployment of digital twins, ethical and privacy issues, the economics of MDT use on a large scale, and health equity issues, among many others. Another topic, vitally important for digital twin deployment, is uncertainty quantification. There are many contributors to model uncertainty, including the stochasticity that many models, in particular ones that involve the immune system, display. This too was not discussed at length at the Forum for lack of time. Most of these topics are addressed in both the EDITH draft strategic plan^8^ and the National Academies report^11^, and we encourage the reader to consult them.
